# Exogenous Control of the Expression of Group I CD1 Molecules Competent for Presentation of Microbial Nonpeptide Antigens to Human T Lymphocytes 

**DOI:** 10.1155/2011/790460

**Published:** 2011-03-22

**Authors:** Angelo Aquino, Grazia Graziani, Ornella Franzese, Salvatore P. Prete, Enzo Bonmassar, Laura Bonmassar, Stefania D'Atri

**Affiliations:** ^1^Department of Neuroscience, University of Rome “Tor Vergata”, Via Montpellier 1, 00133 Rome, Italy; ^2^Laboratory of Molecular Oncology, Istituto Dermopatico dell'Immacolata-IRCCS, Via dei Monti di Creta 104, 00167 Rome, Italy

## Abstract

Group I CD1 (CD1a, CD1b, and CD1c) glycoproteins expressed on immature and mature dendritic cells present nonpeptide antigens (i.e., lipid or glycolipid molecules mainly of microbial origin) to T cells. Cytotoxic CD1-restricted T lymphocytes recognizing mycobacterial lipid antigens were found in tuberculosis patients. However, thanks to a complex interplay between *mycobacteria* and CD1 system, *M. tuberculosis* possesses a successful tactic based, at least in part, on CD1 downregulation to evade CD1-dependent immunity. On the ground of these findings, it is reasonable to hypothesize that modulation of CD1 protein expression by chemical, biological, or infectious agents could influence host's immune reactivity against *M. tuberculosis*-associated lipids, possibly affecting antitubercular resistance. This scenario prompted us to perform a detailed analysis of the literature concerning the effect of external agents on Group I CD1 expression in order to obtain valuable information on the possible strategies to be adopted for driving properly CD1-dependent immune functions in human pathology and in particular, in human tuberculosis.

## 1. Introduction

Cell-mediated immunity involved in host resistance against *mycobacteria* and other infectious agents appears to rely to a large extent on classical HLA-restricted responses against microbial peptides [1] mediated mainly by interferon (IFN) *γ*-producing T-cells [2]. However, in recent years growing attention has been given to T-cell-mediated responses directed against lipid or glycolipid antigens presented by four relatively nonpolymorphic CD1molecules ([3–5], reviewed in [6]). 

Two groups of CD1 isoforms expressed on the cell membrane of various antigen-presenting cells (APCs) have been identified in the course of the last 20 years. In particular, Group I (i.e., CD1a, CD1b, CD1c) and the isoform CD1e, that is confined to the intracellular compartment and is classified as Group III by some authors, are detectable in man but not in mice. On the contrary, Group II (i.e., CD1d, a biological entity outside the scope of the present review) is expressed in mice and men as well, and is involved in Invariant Natural Killer T-cell responses (specifically reviewed in [7]). The molecular structure of CD1 is similar to that of MHC class I. Both CD1 and MHC class I are comprised of heavy chains of similar length, which are organized into three extracellular domains (*α*1, *α*2, and *α*3) and bind *β*2 microglobulin.

Group I CD1 molecules are expressed most prominently on APCs of the myeloid lineage, including dendritic cells (DCs) derived from circulating monocytes (MOs). Peripheral blood CD1^−^/CD14^+^ MOs can be activated by granulocyte-macrophage colony stimulating factor (GM-CSF) alone or more efficiently in combination with interleukin-4 (IL-4) (i.e., GM-CSF + IL-4, hereafter referred to as G4) to express Group I CD1 glycoproteins [9, 10]. These molecules are the products of the *CD1A*, -*B*, and *-C *genes and are known to be involved in the presentation of nonpeptide microbial antigens to T-cells [6, 10–12]. In particular, Beckman et al. in 1994 [13] discovered that the CD1b-presented antigens obtained from *Mycobacterium tuberculosis* were mycolic acids, that is, lipids associated with microbial cell wall. Later, it was demonstrated that CD1 molecules are competent for presentation of a great variety of microbial antigenic lipid structures to T-cells, so that CD1 could be tentatively considered a wide spectrum system of anti-infectious immune surveillance [6].

Particular attention of the present review is dedicated to the studies concerning the CD1 system predominantly engaged in antitubercular responses, and therefore involved in mycobacterial lipid presentation to CD1-restricted T-cells. A fraction of responder T-cells comes from the CD4**^−^**/CD8**^−^** phenotypic subset of CD3**^+^** T-cell receptor (TCR) *α*/*β* T-cells. These cells, sometimes referred to as double-negative TCR *α*/*β* T lymphocytes [14], proliferate and generate cytotoxic clones following interaction with mycobacterial glycolipids, presented by CD1b**^+^** DCs-derived from G4-preactivated MOs. However, CD1-restricted CD8**^+^** or CD4**^+^**TCR *α*/*β* T-cell clones [15, 16] and TCR *γ*/*δ* T-cells [3, 17] have also been demonstrated. Thus, responder cells that potentially play a role in CD1-restricted responses to nonpeptide antigens, have been found to belong to all of the major phenotypic subsets of T-cells. Noteworthy is the general observation that CD1-restricted recognition of bacteria-associated lipids results in killing of the infected cells as well as of the microorganism, thus providing presumably a way to prevent infection spreading in the host [15, 18].

The induction of effector T-cells against microbial antigens is accompanied by the presence of autoreactive CD1-restricted T-cells directed against self-lipid antigens [19]. These lymphocytes appear to cooperate in early suppression of invading microorganisms, in the induction of CD1-restricted memory T-cells and in the maturation of DCs able to produce substantial amounts of IL-12. In turn, IL-12 stimulates T-cells to produce IFN*γ* (reviewed in [20]) and plays an important role in antitubercular immunity [21]. Autoreactive CD1-restricted T-cells have also been accused to take part in the immune mechanisms underlying multiple sclerosis (MS) and Guillan-Barre syndrome [22, 23]. However, detection of autoreactive cytotoxic T lymphocytes in patients affected by autoimmune disease, does not necessarily mean that these cells play a role in the pathological events affecting target organs.

Up to now, it has not been definitely established whether tuberculosis prevention could be achieved through vaccinial procedures based on *M. tuberculosis*-associated lipids as sensitizing agents. Improvement in the course of the disease has been noted in guinea pigs sensitized with lipid extracts of *M. tuberculosis *[24, 25]. Moreover, a recent study published by Felio et al. [26] showed that human Group I CD1 transgenic mice are competent for mounting a CD1-restricted adaptive immune responses to *mycobacteria*, thus allowing further preclinical investigations on lipid-based antitubercular vaccines in mouse models.

**Figure 1:**
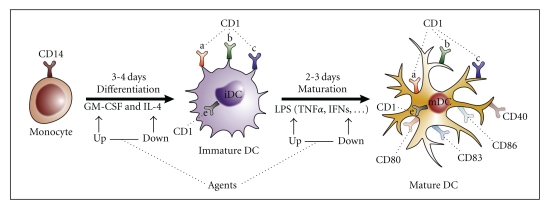
*Dendritic Cell (DC) generation and maturation.* Schematic drawing depicting the differentiation of monocytes to immature DC (iDC), generation of mature DC (mDC) and cytokines involved in these processes. Dotted lines point to the modulating effects of external agents.

**Figure 2:**
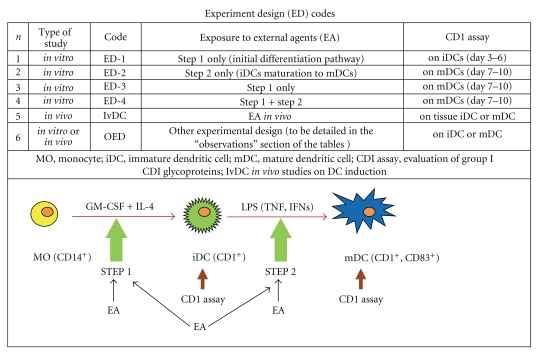
Effect of external agents on group I CD1 glycoprotein expression.

In view of a potential role of Group I CD1 glycoprotein-dependent presentation of mycobacterial lipids to T-cells, it is reasonable to hypothesize that pharmacological or biological agents able to modulate CD1 expression could modify host's responses against infectious diseases, including infections caused by *M. tuberculosis.* Therefore, the aim of the present short survey is to illustrate the data presently available in the literature, relative to the influence that can be exerted by external agents on Group I CD1 molecule expression. In particular, the reported studies will consider human MOs driven *in vitro *or *in vivo* to differentiate into immature and thereafter mature DCs (Figures 1 and 2) competent for peptide or nonpeptide molecule presentation to T-cells.

## 2. In Vitro and In Vivo Assays of CD1 Induction

 A classical experimental design to explore the functional pathways involved in the differentiation and maturation of human myeloid DCs *in vitro* system, starting from purified CD14**^+^** MOs obtained from peripheral blood mononuclear cells (PBMNC), can be described as follows (Figure 2):

**Step 1**

*In vitro* cultivation of MOs with G4 for 3–6 days (or, in some cases, for up to 7 days). This treatment is able to induce “immature DCs” (iDCs) showing high expression of CD1a, CD1b, and CD1c glycoproteins on cell membrane, competent for lipid antigen presentation to CD1-restricted T-cells.

**Step 2**

*In vitro* culture of iDCs with lipopolysaccharide (LPS) and/or various cytokines (e.g., TNF*α*, IFN*α*, TGF*β*, etc.) for additional 2-3 days, leading to mature CD83^+^ DCs (mDCs), fully competent to behave as classical APCs.

In a large number of studies published in more than 15 years, iDCs have been also generated from cord blood CD34**^+^** cells cultured *in vitro* with a cocktail of cytokines containing GM-CSF. In addition, several investigations have been conducted *in vivo* by evaluating the number of DCs in various organs, in different clinical and treatment conditions using immunohistochemical detection of mainly CD1a^+^ cells.

All these methods, able to explore the functional pathways leading to mDCs, allowed to test the effect of a number of exogenous agents on the expression of Group I CD1 molecules induced in host's cell population involved in resistance against pathogens, including *mycobacteria*.

In order to offer a concise picture on the external control of CD1 expression, the present review provides information on the complex relationship between *mycobacteria* and CD1 levels, and four tables summarize schematically what we presently know on the regulation of CD1 expression by pharmacological and biological agents. Moreover, with the intent to provide a simplified information on the experimental strategy utilized for studying the influence exerted by exogenous agents on CD1 expression during myeloid DCs induction and maturation, we decided to adopt the codes that are illustrated in Figure 2.

## 3. CD1 Expression

It is generally agreed that transcriptional control of gene expression and posttranscriptional regulation of mRNA function are usually under the control of proteins targeting specific DNA sequences (i.e., transcription factors) and microRNAs, respectively. In particular, expression of Group I* CD1* genes is under the control of transcription factors, that have been described in detail for CD1a glycoprotein by Colmone et al. [27]. A minimal 1000-bp region upstream of the translation start site has been identified as necessary for proximal promoter activity required for *CD1A *transcription. This region contains multiple sites that were considered to be coordinatively involved in* CD1A* gene expression on the basis of a series of experiments performed by means of deletion and site-specific mutant analysis. In particular, a critical role appeared to be played by a potential cAMP response element (CRE), 965 bp upstream of the *CD1A *translation start site. It was found that the CRE-binding protein 1 (CREB-1) and the activating transcription factors-2 (ATF-2) that are enlisted among the ATF/CREB family members, are able to bind this site *in vitro* and *in vivo *in various cell types, including human MOs [27]. Moreover, the results of these studies speak in favour of ATF-2-induced inhibition counterbalanced by a stimulatory activity on gene transcription by CREB-1, possibly through a competition of CREB-1 and ATF-2 for CRE binding. The hypothesis of opposite control performed by two transcription factors acting on the same gene promoter appears to be supported by the studies published by Niwano et al. [28] who proposed a similar mechanism for endothelial nitric oxide synthase.

In the present survey of the literature, we noticed the emerging role played by miRNAs on hematopoiesis (reviewed in [29]). Therefore, we have considered the possibility that miRNAs could affect *CD1* expression. An *in silico* analysis was performed using the miRanda (http://www.microrna.org/) and TargetScan (http://www.targetscan.org/) algorithms for miRNA target prediction. Under miRanda analysis, miRNA list indicates conserved miRNAs with good mirSVR scores [8]. As illustrated in Table 1, this analysis revealed that mRNAs transcribed from all three Group I* CD1 *genes can be targeted and potentially regulated at the 3′UTR region by a number of different miRNAs. In particular, 10 miRNAs have been found to share a potential capability of controlling the transcriptional activity of two CD1 genes. Six miRNAs (i.e., 33a, 33b, 421, 495, 590-3p, and 590-5p) could target both CD1a and CD1c, whereas miRNA-224 could be active on CD1a and CD1b, and 3 miRNAs (i.e., 129-5p, 185 and 203) appear to be theoretically competent to target CD1b and CD1c. However, up to now no study able to validate the *in silico *prediction patterns is available from the literature. Nevertheless, a number of miR genes have been found to be involved in the regulation of immune responses [30, 31] and acute inflammation [32]. Moreover, quite recently Kuipers et al. [33] described that microRNAs control maturation, function, and maintenance of DCs in the epidermis (i.e., Langerhans cells, LC) *in vivo*. In addition, exchange of genetic material between prokaryotic and eukaryotic multicellular organisms has been described [34]. Therefore, since pathogenic microorganisms, including *mycobacteria *contain a large amount of small noncoding RNA [35, 36], it is reasonable to hypothesize that invading microbes could control gene expression of host eukaryotic cell through their miRNA-like molecules to acquire a survival advantage.

## 4. Mycobacteria and CD1 Expression

Anti-tubercular immunity relies on humoral and cell-mediated immune responses against *M. tuberculosis*-associated epitopes of various origin, and possibly includes CD1-presented lipid antigens recognized by dedicated T-cell subpopulations [37]. More than eighty years ago, attenuated strains of *M. bovis *(i.e., Bacillus Calmette-Guerin, BCG) were developed and utilized as antitubercular vaccine, since they share a variety of antigenic molecules with virulent pathogenic bacilli [38]. Although BCG vaccine reduces the risk of severe forms of tuberculosis in early childhood, unfortunately it is not very effective in preventing the pulmonary infection in adolescents and adults, the populations with the highest rates of tuberculosis disease. Moreover, *M. tuberculosis* is changing and evolving, making the development of new vaccines [39] more crucial to control the disease that is continuously expanding, favored, at least in part, by AIDS pandemia.

In the last years, a considerable amount of experimental studies has been dedicated to investigate the complex relationship between the infection with virulent *M. tuberculosis* or BCG and functional activity of the CD1 system. A number of studies confirm that lipid antigens recognized and presented by Group I CD1 glycoproteins include fatty acids isolated from *M. tuberculosis* cell wall [40]. Among others, they comprise the fatty-acid-derived mycolic acid, the lipopeptide didehydroxymycobactin [41], the isoprenoid-like structure mannosyl phosphomycoketide [42], and the acylated sulfoglycolipid Ac2SGL [43].

In this context, CD1b appears to play a particularly important role, since CD1b-restricted T lymphocytes recognize a large variety of mycobacterial lipids [44], including *M. tuberculosis *Ac2SGL antigens [45]. Moreover, CD1b groove is much larger than that associated with the other CD1 isoforms, so that it can adjust long chain foreign lipids, including long mycobacterial mycolates that are not presented by the other CD1 molecules [46]. On the basis of all these findings and taking into account additional information from the literature (reviewed in [6, 46]), it is reasonable to consider Group I CD1 as a relevant part of the complex antigen-presenting systems involved in the T-cell-dependent immune response machinery against *mycobacteria*. Actually, in human leprosy lesions CD1 expression correlates with host immunity as manifested by active cellular immunity to *M. leprae* [47]. A number of clinical and experimental data indicate that long-lived immunity to *M. tuberculosis* relies largely on antigen-specific CD4^+^ and CD8^+^ T-cells that could play consistent roles in vaccination strategies [48]. Therefore it is reasonable to hypothesize that CD1-restricted effector T lymphocytes, that show a limited repertoire but are able to recognize large amounts of lipid antigens based on antigenic cross-reactivity [49], would contribute to antitubercular immunity. Ulrichs et al. [50] collected PBMNC from patients with pulmonary tuberculosis, from asymptomatic individuals with known contact with *M. tuberculosis *documented by conversion of their tuberculin skin tests, and from healthy tuberculin skin test negative subjects. *In vitro*, in presence of autologous CD1^+^ iDCs, the extent of CD1-restricted T-cell responses to a lipid extract of* M. tuberculosis *was tested by means of proliferation and IFN*γ* production by effector T-cells. The results showed that T-cells from asymptomatic *M. tuberculosis-*infected donors were significantly more responsive than those obtained from uninfected healthy donors. Moreover, essentially no CD1-restricted T-cell response was detectable in lymphocytes collected from patients with active tuberculosis prior to chemotherapy. However, significant antilipid immune reactivity became detectable in blood samples drawn two weeks after the start of treatment, as a possible consequence of chemotherapy-induced relief of the inhibitory effect exerted by *mycobacteria* on cell-mediated immunity [51].

In order to better define the possible role that can be played by CD1-dependent antimycobacterial immunity, it is important to identify the target of CD1-restricted effector T-cells and the modality of target suppression. Of note are the findings illustrated by Vincent et al. [52] who used CD1-restricted human *α*/*β* T-cells generated by autologous DCs in presence of microbial detergent extracts from *M. tuberculosis*, *E. coli*, or *Y. enterocolitica*. Effector T-cells were found to be active in terms of proliferation and IFN*γ* release when tested against target cells presenting microbial lipid antigens via CD1a, CD1b, or CD1c molecules. However, similar activity, although to a lower extent, was detected in absence of foreign lipids, thus indicating that sensitized lymphocytes were also endowed with effector function against self-lipids. The authors propose that CD1-restricted T lymphocytes fit in two T-cell populations, that is, naive T lymphocytes able to mount an adaptive response to microbial lipids as well as memory/effector T-cells. The latter population, characterized by reactivity against self and foreign lipids, would be particularly dedicated to rapid initial immune responses against invading pathogens and yet able to undergo clonal expansion responsible for long-standing cellular memory to foreign lipid antigens. Actually, Nguyen et al. [53] have recently reported that upon experimental vaccination of cattle, CD1b-restricted memory T-cell response can be elicited by the mycobacterial glycolipid glucose monomycolate. 

The effector function of T lymphocytes against microbial targets, including *M. tuberculosis *follows a rather complex pattern (reviewed in [20]). When primed T-cells interact with CD1^+^ *mycobacteria*-infected target cells, they kill directly* mycobacteria* through granulysin/perforin-based mechanism release [54], or they induce Fas-dependent apoptotic death of target cells without killing the intracellular infectious agent. In this case *mycobacteria *are released and infect adjacent macrophages and DCs where invading bacilli are possibly killed, depending on microbial burden. In addition to direct cytotoxic effects, CD1-restricted T-lymphocytes release Th1 cytokines (i.e., IFN*γ* and TNF*α*) that activate the microbicidal functions of macrophages and DCs [20]. 

Recently, the role of IFN*γ* released by CD1-restricted effector T-cells has been subjected to detailed analysis by Lee and Kornfeld [55]. These authors reported that IFN*γ* released by T-cells inhibits bacterial replication in infected macrophages carrying low intracellular burden of* mycobacteria*, thus contributing to host defenses against tuberculosis. However, when macrophages are engulfed with high bacteria load, IFN*γ* facilitates host cell death, thus promoting necrosis and spreading of the infection, with potentially adverse effects on the course of the disease. 

A large body of experimental data is presently available from the literature showing that *mycobacteria* have developed highly sophisticated strategies to escape host's resistance based either on innate or adaptive immunity (reviewed in [56]). Tuberculosis is predominantly a lung disease characterized by long chronic course due to persistent and sometimes dormant infection. It is well documented that upon contact with inhaled *M. tuberculosis,* both alveolar macrophages, that do not express CD1 molecules, and CD1^+^ DCs phagocytose *mycobacteria. *But most of the microorganisms are taken up by macrophages that are by far more efficient than resident lung DCs in the ability to phagocytose and possibly kill bacteria [57]. However, the fate of *M. tuberculosis* within the infected alveolar macrophage depends on the state of activation of the phagocyte. Actually, the bacillus is able to survive preferentially within a macrophage subpopulation displaying an anti-inflammatory phenotype with a reduced oxidative burst. Moreover, phagocytosed *mycobacteria* end up in a phagosome, the maturation of which is arrested at an early stage [58], at least in part by *mycobacteria*-released glycolipids, such as lipoarabinomannan and phosphatidylinositol mannoside [59]. *M. tuberculosis* inhibits phagosomal acidification, prevents phagosome-lysosome fusion and survives within macrophages by avoiding lysosomal delivery thanks, at least in part, to coronin 1 that is actively recruited to mycobacterial phagosomes [60]. Since alveolar macrophages do not express CD1 molecules, and mycobacterial peptide antigens confined to phagosomes are excluded from the classical MHC-I presentation pathway, they cannot be targeted by MHC-I- or CD1-restricted cytotoxic lymphocytes. Therefore, in the lung environment, host's defenses against* mycobacteria* are mainly activated through apoptosis induction of infected alveolar macrophages followed by cross-priming of resident DCs endowed with the appropriate machinery for peptide and lipid/glycolipid antigen presentation to T-cells [61]. However, mycobacterial infection inhibits specifically macrophage apoptosis [62], thus preventing DC cross-priming and consequently providing an additional mechanism of impairment of host's T-cell defenses based on bacterial antigen recognition.

Infection with *M. tuberculosis *can also adversely affect DC function by interfering with their expression pattern of antigen-presenting molecules. Therefore, among the different escape mechanisms operated by* mycobacteria*, of particular relevance for the present survey are the complex autocrine and paracrine devices that the microorganism uses to control the induction of Group I CD1 molecule expression in infected and adjacent noninfected MOs. In 1998 Stenger et al. [63] exposed *in vitro* MOs from healthy donors to G4 for 3 days, obtaining iDCs expressing high levels of Group I CD1 glycoproteins. Thereafter, iDCs were heavily infected with *M. tuberculosis* that was able to suppress entirely CD1 expression within 24 h independently from any cytokine intervention. On the other hand, Prete et al. [64] reported later that *in vitro* coculture of BCG with untreated MOs was able to induce GM-CSF release by infected cells leading to limited CD1b expression. Modest upregulation of Group I CD1 antigen expression was also described by Roura-Mir et al. [65] in untreated MOs after* in vitro* infection with *M. tuberculosis* at 2 or 10 bacteria per cell. These authors report that their findings could be explained, at least in part, through Toll-like receptor-2 (TLR-2) signaling induced by mycobacterial cell wall lipids. A possible, although limited induction of CD1 expression by *mycobacteria *has also been described *in vivo*. Videira et al. [66] found that prophylactic administration of intravesical BCG to prevent tumor recurrence in bladder cancer patients, was followed by upregulation of *CD1A*, *CD1B, CD1C,* and *CD1E* gene transcripts in cells obtained from urothelium biopsies. This effect was significantly higher in patients with a more favorable response with respect to that observed in patients with early tumour recurrence [66]. Marked accumulation of CD1a**^+^** LC after mycobacterial stimuli was also described in leprosy skin lesions [67]. On the other hand, *in vitro* maturation of MOs to CD1a**^+^** DCs under the influence of G4 and LPS was found to be sensibly impaired when MOs were collected from patients with pulmonary tuberculosis [68]. The intriguing Janus-like behavior of *mycobacteria* relative to CD1 expression has been investigated in 2001 by Prete et al. [69] and Giuliani et al. [70], who found that BCG induced *in vitro* a limited expression of CD1 in untreated MOs from healthy donors, but inhibited markedly G4-induced CD1 upregulation in the same cells. Thereafter, further investigations confirmed that *in vitro *infection with *mycobacteria* downregulates CD1 expression [71, 72]. In particular, upon exposure to G4, MOs infected with *M. smegmatis *failed to express CD1a and evolved directly into CD83**^+^** mDCs [73]. In 2007, Prete et al. [74] provided direct experimental evidence that *in vitro *exposure of healthy MOs to BCG induced release of both GM-CSF and IL-10, and that the interplay between the two cytokines was presumably involved, at least in part, in the Janus-like behavior of BCG. Actually, early GM-CSF release was responsible for the limited autocrine and paracrine CD1 induction. On the other hand, slightly delayed appearance in culture medium of IL-10 produced by BCG-infected MOs contributed to the severe limitation of further increase of CD1 proteins, even in the presence of exceedingly high concentrations of added GM-CSF. More recently, Gagliardi et al. [75] reported that *mycobacteria* trigger phosphorylation of p38 mitogen-activated protein kinase (p38 MAPK) in human MOs, leading to CD1 expression impairment. In fact, pretreatment with a specific p38 MAPK inhibitor allows infected MOs to differentiate into CD1**^+^** DCs, which are fully capable of presenting lipid antigens to specific T-cells. Further studies have been conducted on the possible role of cytokines in restraining the GM-CSF-induced upregulation of Group I CD1 glycoproteins in* mycobacteria* infected MOs. Quite recently, Remoli et al. [76] confirmed the results of the studies described by Prete et al. [74] showing that IL-10 produced by MOs infected with* M. tuberculosis* is responsible for *in vitro* suppression of CD1. Moreover, consistently with the results obtained previously by the same group [75], they suggested that IL-10 release by infected MOs was induced by the activation of p38 MAPK signal transduction pathways. Several reports from the literature indicate that *mycobacteria* activate* IL-10 *gene and promote IL-10 release from MOs, phagocytes, and DCs through different intracellular pathways, including PI3K/AKT and p38 MAPK [77–81], phosphorylation and activation of dsRNA-activated serine/threonine protein kinase [82] and glycogen synthase kinase 3 [83]. Noteworthy is the role of proline-glutamic acid/proline-proline-glutamic acid family of proteins of *M. tuberculosis* that can stimulate macrophages to secrete IL-10 via activation of the TLR-2 leading to an early and sustained activation of p38 MAPK, which is critical for IL-10 induction [84]. The role of MAPK in the impairment of CD1 expression by *mycobacteria* has been also confirmed and emphasized very recently by Balboa et al. [85] who found that* mycobacteria*-induced loss of CD1b molecules partially involves TLR-2/p38MAPK activation. 

Several other molecular mechanisms distinct from those relative to impairment of CD1 gene transcription could be involved in *mycobacteria*-induced decrease of CD1 expression or of antigen presentation efficiency. The complex cycle of CD1 biosynthesis, cell surface expression, and lipid loading [12, 44, 86] highlights the several means by which *mycobacteria* can interfere with CD1 expression on cell membrane and antigen presentation to T-cells. After biosynthesis in the endoplasmic reticulum, CD1e remains in the cell, whereas all other CD1 molecules reach the cell surface through the Golgi and trans-Golgi network where they bind to self-lipids. Direct loading of lipids may occur at the plasma membrane, as described for glycosphingolipids that bind to CD1b on the cell surface at neutral pH. Thereafter, glycosphingolipids are recognized without internalization or processing and stimulate specific T-cells [87]. Moreover, various cell-surface CD1a proteins are stabilized by exogenous glycosphingolipids and phospholipids present in serum [88]. 

As a rule, processing and presentation of microbial CD1-bound lipid antigens require that CD1 molecules, loaded with self-lipids, undergo a recycle process. CD1-self lipid complexes are internalized, traffic through the endosomal compartments, where loading and/or exchange with exogenous lipid antigens occur, then the new CD1-nonself lipid complexes re-emerge on plasma membrane. This process resembles peptide sampling by MHC class II proteins, although MHC class II molecules may reach the endocytic compartment directly from the trans-Golgi-network, without first travelling to the cell membrane. 

Cell surface CD1 molecules are internalized according to two distinct mechanisms. Specifically, CD1a molecules, which lack a tyrosine-based internalization motif, are internalized to the early endosomes [89] through a clathrin/dynamin-independent manner and recycle back to the plasma membrane through a mechanism that relies on small GTPases, such as Rab22 and ADP-ribosylation factor 6. Both CD1b and CD1c molecules, instead, have a tyrosine-based motif in their cytoplasmic tail and are internalized through clathrin-coated pits via the adaptor protein 2 (AP-2). Thereafter, CD1b is transported to the late endosomes and, after binding to AP-3, traffics to the lysosomes and then recycles to the plasma membrane. On the other hand, CD1c, after reaching the sorting endosomes, routes to the early endosomes, and, although to a lesser extent, to the late endosomes and lysosomes, and then recycles to the plasma membrane. It follows that CD1c operates a comprehensive survey for lipid antigens throughout the endocytic system [90].

The entire CD1 recycling pattern reveals that a large variety of molecular targets could be affected by *M. tuberculosis. *In addition to that, it must be considered that intracellular lipid loading presumably requires the functional intervention of a number of helper and adaptor molecules, including saposins and apolipoproteins [91, 92] and CD1e itself [93, 94]. Moreover, acidic pH promotes lipid binding to CD1b proteins, thus suggesting that pH fluxes during endosomal recycling regulate the conformation of the CD1 heavy chain to control the size and rate of antigen capture [95]. Within this context, it is worth of note the finding that *mycobacteria *impair phagosome acidification [58] thus reducing the extent of mycobacterial lipids bound to CD1b for T-cell presentation.

## 5. HIV and CD1 Expression

Interestingly enough, not only the *mycobacterial* infection, but also HIV or HTLV-1 infection or intracellular presence of HIV products are able to interfere with CD1 expression. For example, HIV-1-Nef was found to interfere with the intracellular trafficking of CD1a [96], although recombinant Nef added to iDCs increases CD1a expression [97]. Moreover, it must be pointed out that viable HIV-1 particles infect target CD4**^+^** T-cells via CD1b**^+^** exosomes [98]. On the other hand, in 30 to 45% of HIV-infected white and African subjects, peripheral blood MOs exposed* in vitro* to G4 followed by LPS gave rise to CD1a^−^ mDCs releasing IL-10 but not IL-12 [99]. In addition, DCs from HTLV-I-infected monocytes fail to present adequate amounts of CD1a glycoprotein [100]. 

Preliminary investigations of experimental design (ED)-1 type (see ED codes illustrated in Figure 2) performed in our laboratory, revealed also a possible link between HIV infection and CD1 system, presumably relevant to the increased susceptibility of HIV-infected individuals to *mycobacteria*. A vector expressing *tat* DNA (PCV-TAT, [101]) under the control of the major adenoviral late protein, and a control empty vector (PCV-0) were kindly provided by Barbara Ensoli MD of the Italian National Institute of Health. Peripheral blood MOs of healthy donors were incubated with G4 alone or with G4 + a supernatant obtained from the human T-cell leukemia line Jurkat transfected with PCV-0 (sup-PCV-0) or with PCV-TAT (sup-PCV-TAT). The results of a representative experiment demonstrated that *tat*-induced factors released by transfected cells are able to down-regulate CD1b expression. In fact, after 5-day exposure to G4 *in vitro*, iDCs generated in the absence of supernatants or in the presence of sup-PCV-0 showed 72% and 79% CD1b^+^ cells, respectively. In contrast, when iDCs were generated in the presence of sup-PCV-TAT, the percentage of CD1b**^+^**cells dropped significantly to 54% (Franzese et al., in preparation). Moreover, if monoclonal antibodies against IL-10 were added to G4 + sup-PCV-TAT at the onset of iDC generation, the percentage of CD1b^+^ cells raised to 81%. These results along with previous findings indicating that TAT induces IL-10 in MOs [102] and that IL-10 downregulates CD1 expression [74–76, 103–106], are consistent with the hypothesis that IL-10, generated in the presence of TAT, plays a critical role in compromising CD1b expression.

## 6. Chemical, Biological, and Physical Agents Affecting CD1 Expression

### 6.1. Drugs

A number of natural and synthetic compounds of pharmacological interest are able to modulate the expression level of Group I CD1 proteins on immature and/or mature DCs, either *in vitro* and *in vivo*, as reported in Table 2.

As expected, most of the immunosuppressant and anti-inflammatory agents, including corticosteroids, nonsteroidal anti-inflammatory drugs (NSAID), and anti-asthma compounds, down-regulate cytokine-induced CD1 expression of MOs and impair their functional activity. However, local application of Pimecrolimus on skin in atopic dermatitis, is followed by increase in the number of CD1a^+^ cells. Moreover, *in vitro* exposure of CD34^+^ peripheral blood progenitor cells to Tacrolimus favors the expression of CD1a induced by 14-day treatment with cytokines. Notable exceptions to the inhibitory effects of anti-inflammatory drugs is also represented by Piceatannol (a stylbene compound similar to resveratrol) and terpenes that were found to increase CD1a expression after G4 treatment *in vitro* of MOs obtained from healthy donors. Of sensible relevance to the problem of MS therapy and identification of disease pathogenesis is the finding that Glatiramer acetate (GA), alone or in combination with IFN*β*, is able to down-regulate CD1 expression *in vitro* or *in vivo*. Similar inhibitory effects have been described *in vitro* with vitamin D3 that shows beneficial effects in MS management. These observations appear to provide further support to the hypothesis that significant participation of CD1-restricted T-cell responses against self lipid antigens is involved in the neuronal damage occurring in MS.

Among chemotherapeutic agents, antitubercular (rifampicin) or antiretroviral (entecavir) drugs tend to up-regulate CD1 expression, whereas zidovudine (AZT), that inhibits iDC proliferation, diminishes the overall availability of CD1a^+^ cells. In the area of antineoplastic therapy, reduction of cytokine-induced CD1 levels by various agents is the dominant finding, as shown *in vitro *by histone deacetylase (HDAC) inhibitors, tyrosin kinase inhibitors (i.e., imatinib and sorafenib) and antiestrogens, and *in vivo* by thalidomide in multiple myeloma (MM) patients.

More difficult to interpret is the activity of a classical agent largely utilized in mood disorders including bipolar affective disorders, such as lithium. The drug downregulates the *in vitro *cytokine-induced CD1a expression in MOs of healthy donors. However, limited CD1a expression is elicited by G4 in MOs collected from patients with bipolar disorders. In this case, *in vivo* treatment of donor patients with lithium restores full responsiveness of their MOs to G4 exposure *in vitro.*

### 6.2. Cytokines and Autacoids

Table 3 illustrates the limited information available from the literature on the effect of prostaglandins and serotonin on CD1a expression in different experimental conditions *in vitro*. In all cases, the agents show suppressive activity.

When cytokines are considered, GM-CSF and IL-4 are not enlisted in Table 3. Actually, this cytokine combination is used by most of *in vitro* tests, to induce iDCs that express high levels of CD1 proteins (Figures 1 and 2). In particular, GM-CSF is the most potent inducer, whereas IL-4 reinforces the effect of GM-CSF but is scarcely active if used alone. 

A number of data from the literature is presently available on IFNs that show predominant inhibitory effects on CD1 system. While IFN*α* can be involved in the transition from iDCs to mDCs (Figure 1), IFN*β* downregulates CD1 protein expression either *in vivo* or *in vitro*. In addition this cytokine was found to reduce the functional activity of mDCs. Since IFN*β* has acquired a definite role in MS treatment, these results add further support to the hypothesis of the involvement of CD1 system in MS pathogenesis.

Consistent inhibitory effects on CD1 expression are manifested by IL-6 and IL-10 in various experimental conditions. It must be pointed out that in many cases down-regulation of G4-induced CD1 expression provoked by various agents appears to be mediated by the release of IL-6 and more frequently by the release of IL-10 that operates according to an autocrine pattern.

Of interest, finally is the mechanism by which TGF*β* appears to maintain CD1a expression on LC generated *in vitro* from purified CD34^+^ cells. In this case, the expression of CD1a, that is normally found to be elevated in immature LCs, declines with LC maturation. Since TGF*β* prevents LC maturation, it allows the long-term presence of high CD1a levels in LCs.

### 6.3. Biological and Physical Agents

With the exception of the placental growth factor, all biological and physical agents illustrated in Table 4 provoke down-regulation of cytokine-induced CD1 protein expression. The mechanism underlying the effect of various lipids including some contained in human serum, indicates a common target consisting in peroxisome proliferator-activated receptor (PPAR)*γ* that appears to be activated by these molecules in various experimental conditions. The observation that human serum, either for the presence of different lipoproteins or for the presence of IgG and *β*2-microglobulin (Table 4), provides inhibitory effects, poses undoubtedly the question of the efficiency of the CD1 system *in vivo* in infected patients. 

Of considerable interest is the finding that various supernatants of human tumor cell cultures contain inhibitory factors. Although mycoplasma contamination of cultured cells could be, at least in part, responsible for these findings (see Table 5), it cannot be excluded that this type of suppression of antigen-presenting function could be of relevance in tumor-induced immune suppression.

The *in vivo* impairment of CD1a expression by ultraviolet light is not surprising, since the general immune-suppressive effects of this type of radiation has been demonstrated in different effector functions of the immune system.

### 6.4. Infectious Agents or Microorganism Products

*In vitro* and *in vivo* studies concerning modulation of CD1 system by bacterial and chlamydial infections generally demonstrated a CD1 upregulation (Table 5). It is reasonable to speculate that, in certain experimental conditions, TLR-2 activation by microorganisms could be involved [29]. Surprisingly, however, is the finding that antral biopsies performed in* H. pilori*-infected children reveal CD1a/b upregulation respect to normal subjects, whereas *in vitro* exposure of MOs to formalin-killed *H. pilori* prevents CD1 induction by G4. 

Of particular note is the finding that CD1a is up-regulated *in vitro *by G4 more vigorously in MOs obtained from MS patients bearing an infectious disease, with respect to MOs obtained from noninfected MS patients. This observation has been put in relationship with the clinical finding that subjects affected by MS are at particular risk of relapse in the course of bacterial infections. Again, this seems to provide support to the hypothesis of a significant role that could be played by CD1 system in MS. 

Differently from the *in vivo *and *in vitro* effect of the bacteria and chlamydia reported in Table 5, infections with various protozoa, with at least two types of helminthes, and viruses such as HHV-8 and Cytomegalovirus leads to impairment of CD1 expression in various types of experimental design. This is not surprising since the general immunodepressive activity of these infections has been known for several years.

When microorganism products are considered, only attenuated Dengue-2 live vaccine, malaria-associated AMA-1, and staphylococcus superantigen are able to up-regulate cytokine-induced CD1 expression. Toxins and malaria hemozoin provide opposite effects on the system. A particular feature that distinguishes the activity of pertussis toxin from the other microorganism products resides in its unusual property of suppressing CD1a expression selectively, without reducing the levels of the other components of the system (i.e., CD1b and CD1c). It is not excluded that this could allow selective analysis of *CD1A* gene regulation distinct from that of the other *CD1* genes. 

Finally, of relevance is the finding that LPS is able to down-regulate G4-induced CD1a. LPS, that is considered the standard agent for generating mDCs from iDCs (Figures 1 and 2), is a common constituent of pathogenic or nonpathogenic microorganisms, being present in the cell wall of gram-negative bacteria. Therefore, it is reasonable to consider that this molecule could play a significant role in the clinic, possibly through its modulating activity on CD1 expression and DC maturation.

## 7. Conclusions and Perspectives

Fine tuning of biological functions governed by a complex signaling network is commonly seen in living organisms, and the CD1 system does not represent an exception to this rule. This opens up several options to intentionally manipulate the CD1 expression in order to enhance or depress antigenic lipid presentation according to the therapeutic needs. The results of the literature analysis presented here clearly demonstrate that a large variety of different externally acting agents, either of synthetic or natural origin, can affect profoundly the expression levels of CD1 glycoproteins, with a possible consequence on DC-mediated lipid presentation to T-cells. Actually, Group I CD1 glycoproteins are mainly involved in the presentation of *M. tuberculosis*-derived lipids to CD1-restricted T-cells. Pharmacological amplification of the system could provide a significant help for vaccination and treatment modalities concerning millions of subjects presently exposed to tuberculosis threat. In particular, the rapidly expanding area of small RNAs capable of controlling directly or indirectly the expression level of an extremely high numbers of genes, could be carefully considered for planning new types of antimycobacterial vaccines. It is reasonable to predict that properly designed siRNA(s) could be combined in a near future, with BCG or BCG-like vaccines in order to obtain *gene silencing vaccines* able to inactivate the intracellular signals responsible of Group I CD1 protein suppression.

## Figures and Tables

**Table 1:** miRNAs with putative binding sites in the 3′UTR of *CD1A*, *CD1B,* and *CD1C* genes.

Gene	Algorithm	
	miRanda^a^	Target Scan^b^
*CD1A*	19a, 21, 28-5p, 31, 33a, 33b, 146a, 146b-5p, 214, 217, 361-5p, 383, 421, 448, 495, 590-3p, 590-5p, 708, 873	**21**, 28-5p, **31**, **33a**, **33b**, 125a-3p, **138**, **146a**, **146b-5p**, 197, **205**, 224, 421, 448, 495, **590-5p**, 708

*CD1B*	129-5p, 137, 185, 203, 224, 543	**129-5p**, **137**, **203**, 543

*CD1C*	26a, 26b, 33a, 124, 125a-5p, 125b, 129-5p, 181a, 181b, 181c, 181d, 190, 190b, 203, 216a, 216b, 218, 219-5p, 300, 326, 330-5p, 340, 367, 376a, 376b, 381, 410, 421, 433, 455-5p, 494, 495, 505, 506, 539, 543, 590-3p, 1297	**22**, **26a**, **26b**, **33a**, **33b**, **124**, **125a-5p**, **125b**, **129-5p**, **132**, 185, **190b**, **203**, **212**, **216a**, **216b**, **218**, **219-5p**, **221**, **222**, 300, 326, 330-5p, 376a, 376b, 410, 495, **425**, 433, **455-5p**, **489**, 494, 505, **506**, 539, 542-3p, 543, 590-5p, 599, **1297**

^a^Conserved miRNAs with good mirSVR scores [8].

^b^miRNAs broadly conserved among vertebrates (bold) or conserved only among mammals.

**Table 2:** Pharmacological modulation of CD1 molecule expression.

Drug class	Agent	Therapeutic use	CD1^a^	ED^b^	Ref^c^	Observations
Angiotensin receptor antagonists	Losartan (AT1-R) PD123319 (AT2-R)	Hypertension	D U	ED-1 ED-1	[107]	Assay performed on day 7.

Anti-asthma	Suplatast tosilate	Inhibitor of Th-2 responses	D	ED-1 ED-2	[108]	In ED-1 the assay was performed on day 7. DCs were obtained from pts with asthma.

Anti-depressant	Lithium	Bipolar disorders	D	ED-1	[109]	MOs, obtained from bipolar pts, were incubated with G4.
			U	IvDC	[109]	*In vitro* generated DCs from lithium-treated pts showed higher CD1a expression than DCs from untreated pts.
			D	ED-1	[110]	*Mechanism:* CD1 down-regulation is likely mediated through the GSK-3*β* pathway.

Anti-estrogens	Tamoxifen Toremifene	Breast cancer	D	ED-1 ED-2	[111]	Assay performed on day 7.

Anti-inflammatory corticosteroids	Beclomethasone dipropionate (BDP, inhaled)	Asthma	D	IvDC	[112]	In bronchial mucosa of asthmatic pts there is an increase of CD1a**^+^** DCs. Following long-term treatment with BDP the number of CD1a**^+^**DCs went to normal levels of non-asthmatic pts.
	Dexamethasone (DEX)	Inflammatory diseases	D	ED-1 ED-4	[113]	Strong CD1a down-regulation. *Mechanism*: high IL-10 via Extracellular signal-regulated kinases (ERK) phosphorylation.
			D	OED	[114]	CD34**^+^** cord blood stem cells were cultured with SCS, Flt3-ligand and GM-CSF (Pre-DC). After 5 days, TNF*α* and IL-4 were added (differentiation stage). On day, 10 CD-40 ligand and anti-human CD40-ligand were also added (maturation stage). DEX, added during differentiation stage, suppresses CD1a at the end of the immature (day 10) and at the mature stage (day 12). On the contrary, CD1a was expressed at normal levels when DEX exposure was limited to the 2 day maturation stage.
				ED-1 ED-3 ED-4	[115]	The MO-derived DCs were obtained from neonatal cord and adult blood.
	Various including DEX		NC	OED	[116]	CD1a**^+^** cells derived from bronchoalveolar lavage showed lower APC function if treated with DEX *in vitro*.
Anti-tubercular agents	Rifampicin		U	ED-1	[117] [118]	Test on CD1b: the drug does not affect the functional activity of the T-cell clone capable of recognizing the mycolic acid of *M. tuberculosis *origin, presented by CD1b proteins. Test on CD1b: effect obtained at clinical concentration of the drug.

Antiviral	AZT Entecavir	HIV treatment Hepatitis B treatment	NC U	ED-1 ED-1	[119] [120]	AZT inhibits DC proliferation, thereby reducing the total number of DCs.

Bisphosphonates	Zoledronic acid	Osteoporosis	D	ED-4 ED-2	[121]	Mechanism: possibly via IL-10 induction, antagonized by geranylgeraniol.

Disinfectants	Sodium Chlorate		D	ED-1	[122]	Sodium chlorate reduces GAG sulfation on MO surface. Reduction of sulfated CSB impairs IL-4 mediated DC differentiation and CD1a expression.

HDAC inhibitors	MS-275 Sodium valproate	Antitumor	D	ED-4 ED-1	[123]	Mechanism: NF-*k*B, IRF-3 and IRF-8 inhibition. Possible use in inflammatory and autoimmune disorders.
	Na butyrate		D	ED-1	[124]	The agent prevents CD1 upregulation induced by activation of TLR-2.
Immuno stimulant agents	Imiquimod (imidazo quinoline)	Topical use in squamous cell carcinoma	D	IvDC	[125]	In skin biopsies after topical treatment.
	OK-432	In cancer treatment	U	ED-2	[126]	The maturation step was performed with OK-432 which promotes a higher expression of CD1a in respect to that obtained with LPS.

Immuno suppressive agents	Gold sodium thiomalate (GST)	Rheumatoid arthritis (RA)	D	ED-1 ED-2 ED-4	[127]	DCs were obtained from healthy donors or RA pts. The suppression of DC differentiation and function might explain the *in vivo *effect of GST on RA patients.
	Glatiramer acetate (GA) + minocycline (MIN)	Multiple sclerosis (MS)	D	ED-1 ED-4	[128]	DCs were obtained from untreated and GA-treated MS pts. The possible additive effects of GA and MIN on MO-derived DCs, seem to support the use of such combination therapy in MS.
	GA + IFN*β*	MS	D	IvDC	[129]	MOs were obtained from untreated or treated MS pts and from healthy donors. Combination therapy with IFN*β* + GA resulted in a more pronounced decrease of circulating CD1a compared to monotherapy with IFN*β*.
		MS	D	ED-1	[130]	Assay was performed on day 7. DC were obtained from MS pts. Synergistic effects of GA and IFN*β*.
	Monomethyl-fumarate (MMF)	Psoriasis	D	ED-1 ED-4	[131]	MMF interfered with the MO-derived DC differentiation, resulting in impaired maturation of these cells.
	Pimecrolimus	Atopic dermatitis	NC	ED-1	[132]	No interference with the function of DCs, whereas the activation of effector T-cells was inhibited.
			U	IvDC	[133]	In epidermal cells (biopsy) after topical treatment.
	Rapamycin	Immuno-suppressant	U	ED-1 ED-4	[134]	Reduction of MHC-I, MHC-II and Ag uptake.
	Sinomenine		U	ED-1	[135]	The drug prevents LPS-induced DC maturation.
	Tacrolimus (FK506)		D	ED-1 ED-2 ED-4	[136]	Effect on LPS-induced DCs *in vitro. *
			D	IvDC	[137]	Topical treatment of epidermal CD1a**^+^**, in pts with atopic dermatitis.
			U	OED	[138]	Generation of DCs from CD34**^+^** peripheral blood progenitors obtained by culturing the cells with GM-CSF, TNF*α*, stem cell factor for 14 days. FK506 was added throughout the culture starting on day 0.
	Triptolide	Polycystic Kidney disease	D	ED-1 ED-2	[139]	Suppression of DC differentiation and maturation by triptolide may explain some of its immunosuppressive properties.

Insecticides	Rotenone		D	ED-1	[140]	*Mechanism:* increased levels of reactive oxygen species that seem to trigger the differentiation process of DC.

Multidrug resistance (MDR) protein antagonists	MK571[multi drug resistance protein 1 (MRP1) blocker] PSC833 (P-gp blocker)	Possible use in MDR	D	ED-4	[141]	MRP1 transporter activity is important for DC differentiation.
			NC	OED		Langerhans-like DCs were obtained from human acute myeloid leukemia cell line MUTZ-3, cultured with TGF-*β*1, GM-CSF and TNF*α* for 10 days, and MDR antagonists were added on day 4, 7, and 10.

Monoclonal antibodies	Infliximab	Anti-TNF*α*	D	ED-1 ED-4	[142]	MOs from psoriasis pts. Reduction of antigen-presenting capacity of DCs, proliferation and IFN*γ* release by psoriatic T-cells.

NSAID	Acetylsalicylic acid (ASA)	Inflammation	D	ED-1 ED-4	[143]	The new nitric oxide releasing-ASA (NCX-4040, NCX-4016) did not affect the expression of CD1a during maturation stage (ED-4).
	Niflumic acid (NFA)	Inflammation	D	ED-4	[144]	NFA inhibits LPS-induced DC maturation by inhibiting co-stimulatory molecule expression and IL-12p70 production.

Microsomal triglyceride transfer protein (MTP) inhibitors	BMS212122	Anti-lipid	D	ED-1	[145]	MTP inhibitors down-regulate self as well as exogenous lipid antigen presentation.

NO donors	DEA-NO, SIN-1, DETA-NO		U NC	ED-1 ED-2 ED-1	[146]	The drugs are TNF*α* receptor inhibitors.

Statins	Atorvastatin	Dyslipidemia	U	OED	[147]	MO-derived DCs were obtained from healthy donors. MOs exposed to atorvastatin in combination with IFN*α*, showed an increased levels of CD1a compared to IFN*α* alone.
	Lovastatin		D	ED-2	[148]	DCs were obtained from MS pts. Lovastatin was added after G4, simultaneously with TNF*α*.

TLR agonists	Pam CSK resiquimod (R848)	Immune response modifier	U	OED	[149]	Induction of CD1a**^+^** cells in freshly isolated BM CD34**^+^** progenitor cells cultured with TLR agonists without cytokines.

Tyrosine Kinase Inhibitors	Imatinib	Antitumor	D	ED-1 ED-4	[150]	In ED-1 the assay was performed on day 7. *Mechanism*: NF-*k*B and AKT inhibition.
			D	OED	[151]	*In vitro* effects of imatinib, added to the culture together with different cytokines, on the development of mobilized human CD34**^+^** peripheral blood progenitor cells into DCs.
	Sorafenib	Antitumor	D	ED-1	[152]	In ED-1 sorafenib was added on day 5.
				ED-2		*Mechanism*: P13MAPK and NF-*k*B inhibition.

Various	All trans-retinoic acid (ATRA)	Various	U	ED-1	[153]	In ED-1 ATRA was associated with GM-CSF without IL-4.
	Retinoic acid (Am80)	Various	D	ED-1	[154]	Am80 treatment ameliorated macro- and microscopic damage in dextran sodium sulfate-induced colitis in mice, and suppressed the colitis induced elevation of IL-12.
	Thalidomide	Multiple myeloma (MM)	D	OED	[155]	MOs were obtained from peripheral blood of MM pts treated or not with thalidomide. For *in vitro* DCs generation standard cytokines were used.
		Sarcoidosis	U	IvDC	[156]	In skin biopsies of sarcoidosis pts treated or not with thalidomide.
	Trimethyl psoralen + PUVA	Psoriasis	D	IvDC	[157]	Biopsies of lesional skin were performed in pts with psoriasis, before treatment, after 2 weeks of treatment or at the end of treatment.
	Dehydro- epiandro-sterone		D	ED-1	[158]	Slight down-regulation. The assay was performed on day 7. Reduction of IL-10 (opposite effect respect to DEX).
	Terpenes: Calamenene T-cadinol	Anti-inflammatory, anti-septic	U	ED-2	[159]	
	Terpenes: Epicubenol, Ferruginol	Anti-septic	U	OED	[160]	MOs were cultured with G4, followed by another 2 days with the drugs. Surprisingly both induce IL-10 generating Treg.
	Piceatannol (stilbene derivative)	Anti-inflammatory, immunomodulatory and anti-proliferative	U	OED	[161]	MOs were cultured with G4 for 6 days, followed by another 2 days in the presence of piceatannol alone. On the contrary high concentration of resveratrol, another stilbene derivative, markedly reduces CD1b expression on G4-induced iDCs (Fuggetta et al., in preparation).

Vegetal products	Ginseng saponins (M1 and M4)	Various	U	ED-2	[162]	After G4 DCs were treated on day 6 only with M1 or M4.

Vitamins	Alpha dihydroxy vitamin D3	MS	D	ED-1	[163]	1,25(OH)2D3 hampers the maturation of fully active immunostimulatory MHC-II**^+^**, CD1a**^+^**, CD80**^+^**DCs from MOs.
			D	ED-1 OED	[164]	CD34**^+^** cells were collected by apheresis either from cancer pts after chemotherapy or from healthy donors after G-CSF treatment. For DC generation the cells were cultured with standard cytokines.
			D	ED-2	[165]	DCs were obtained from MS pts. Beneficial action of vitamin D in MS may be associated with its inhibition on both differentiation and maturation of DCs.
			D	ED-4	[166]	Accompanied by overexpression of miR-378 and low expression of miR-155 that could have a role in DC function.
			D	ED-1	[167]	D3 up-regulates colony stimulating factor 1 and downregulates its receptors.
			D	ED-1	[168]	Assay was performed on day 7. Inhibition of DC differentiation and maturation.
	Calcipotriol (vit. D3 analog)	Topical in psoriasis	D	OED	[169]	*In vivo* treated psoriatic skin.

^a^Evaluation of CD1a expression if not otherwise specified: U: upregulation; D: down-regulation; NC: no change.

^b^Experimental design code (see Figure 2).

^c^Reference number.

**Table 3:** Effect of autacoids or cytokines on CD1 molecule expression.

Molecule	CD1^a^	ED^b^	Ref.^c^	Observations
*Autacoids*				

Prostaglandin PGE_2_	D	ED-4	[170]	
	D	ED-1	[171]	Purified CD14**^+^** cells from PB of healthy donors mobilized with G-CSF for allogeneic transplantation.
	D	ED-2		
	LD	ED-3		
	D	ED-4		
	D	ED-1	[172]	
	D	ED-1	[173]	
	D	ED-1	[174]	
Cyclopentenone Prostaglandins (CP) (15d-PGJ2, 12-PGJ2, PGA2, PGD2, and PGE2)	D	ED-1	[175]	MOs + G4 for 7 days. CP were added during the last 24 h of culture without adding maturation factors. In these experimental conditions, CP induced apoptosis.

Serotonin (5-hydroxy-tryptamine, 5-HT)	D D	ED-1 ED-4	[176]	MOs. 5-HT effects mediated via 5-HTR_1/7._ iDCs and mDCs exposed to 5-HT for 24 h did not show alteration of CD1a expression.

*Cytokines*				

IFN*α2*a	D	OED	[177]	MOs cultured for 7 days with (GM-CSF+IL-4+TNF-*α*) *+/*− IFN*α2*a.

IFN*α*2b	D	OED	[178]	MOs cultured with GM-CSF *+/*− IFN*α*2b for 5 days.

IFN*α* + IL-2 or IL-12 alone	NC	IvDC OED	[179]	MOs obtained from PB of pts with renal cell cancer before, during, and after therapy with the indicated cytokines, or from healthy subjects were cultured with G4 for 8 days. The yield of DCs from cancer pts was lower than that from healthy subjects. However, the phenotype of DCs generated from MO of pts was comparable to that of DCs generated from MO of healthy subjects.

IFN*β*1a	D	ED-2	[148]	MOs from PB of untreated pts with MS.
	D	ED-1	[180]	MOs from PB of untreated or IFN*β1*a-treated pts with MS.
	D	ED-4	[181]	MOs from PB of untreated pts with MS. Analysis was performed on CD1a/b/c molecules.
	D	ED-1	[182]	
	D	IvDC	[183]	Evaluation of % of CD1a**^+^**HLA-DR**^+^** MNC in PB of MS pts, either untreated or treated with IFN*β*1a, and in healthy subjects.
	D	ED-4	[184]	Purified CD14**^+^** cells from PB of healthy subjects.

IFN*β1*b	D	ED-1	[185]	MOs from PB of untreated or IFN*β1*a-treated pts with MS and from healthy subjects.

IFN*γ*	D	ED-1	[186, 187]	MOs + G4 for 12 days.

Il-1*β*	NC	ED-1	[188]	
IL-3	U	OED	[189]	CD14**^+^** osteoclast precursors from PB of healthy donors cultured with (M-CSF+ RANKL) +/− IL-3 for 7 days.

IL-6 sIL-6R*α*/IL-6 fusion protein (FP6)	D D	OED	[190]	GPA^−^CD15^−^CD14^−^CD1a^−^IL-6R**^+^** myeloid progenitors (generated after incubation of cord blood-derived CD34**^+^**CD38^−^ cells with SCF+FLT3-L+TPO+IL-3 for 6-7 days) were cultured with (SCF+FLT3-L+TPO+IL-3) +/− IL-6 or FP6 for 11–14 days.

IL-6 sIL-6R/IL-6 fusion protein (FP6)	D D	OED	[191, 192]	CD36^−^CD15^−^CD14^−^CD1a^−^IL-6R**^+^** myeloid progenitors (generated after incubation of cord blood-derived CD34**^+^**CD38^−^ cells with SCF+FLT3-L+TPO+IL-3 for 7 days) were cultured with (SCF+FLT3-L+TPO+IL-3) +/− IL-6 or FP6 for 7 days.

IL-6	D	OED	[193]	Purified CD34**^+^**hematopoietic progenitor cells from PB of G-CSF-treated Pts with MM were cultured with (FLT3-L+TNF*α*+GM-CSF+SCF+IL-4) +/− IL-6 (added on day 0 or day 7 of culture) for 14 days. CD1a evaluation on day 14.
	D	ED-1	[194]	MOs. *Mechanism,* Il-6-induced expression of G-CSF receptor.

IL-10	D	IvED	[195]	Psoriatic skin after systemic IL-10 administration.
	D	OED	[196]	MOs cultured with (GM-CSF+IL-13) +/− IL-10 for 7 days.
	D	OED	[197]	MOs cultured with (GM-CSF+IL-13) +/− IL-10 for 7 days.

IL-13	U	OED	[198]	MOs cultured with G4 or with GM-CSF+IL-13 for 7 days. Higher CD1a upregulation with GM-CSF+IL-13. MOs cultured with G4 for 7 d and then with IL-13 or TNF*α* or IL-4 for 48 h. IL-13 as affective as TNF*α* in inducing maturation of imDC.

Platelet factor 4	D	ED-1	[199]	

TGF-*β*1	U	OED	[200]	Purified CD34**^+^** hematopoietic progenitor cells from cord blood cultured with (FLT3-L+TNF*α*+GM-CSF+SCF) +/− TGF- *β*1 for 10–14 days. Cells generated in the presence of TGF-*β*1 resemble immature LC with high CD1a antigen expression. *Mechanism*: maturation of LC leading to CD1a down-regulation is prevented by elevated E-cadherin expression induced by TGF-*β*1.

^a^Evaluation of CD1a expression if not otherwise specified: D: down-regulation; U: upregulation; LD: limited down-regulation; NC: no changes.

^b^Experimental design code (see Figure 2).

^c^Reference number.

**Table 4:** Effect of biological or physical agents on CD1 molecule expression.

Type of biological agents	Agent	CD1^a^	ED^b^	Ref.^c^	Observations
Growth factors	Placental growth factor (PLGF)	U	ED-2	[201]	Modest upregulation. PLGF antagonizes LPS-induced down-regulation of CD1a in iDCs. *Mechanism*: inhibition of NF-*k*B signal transduction pathway.

Heat-shock proteins	HSP-27	D	ED-1	[202, 203]	*Mechanism: *IL-10 induction.

Immuno-complexes	Anti-OVA rabbit IgG + OVA	D	ED-1	[204]	*Mechanism*: interaction with Fc*γ*RI and Fc*γ*RII.

Ligand proteins	Peptide ligand of melanocortin-4 receptor (NDP-MSH)	D	ED-4	[205]	mDCs from treated precursors show impaired ability to prime T-cells.
	sLAG-3 (CD223) soluble MHC-II ligand	D	ED-1 ED-3	[206]	CD1a down-regulation. *Mechanism * (hypothesis): phosphorylation of PLC*γ*2, p72syk, or AKT molecules.

Lipids	Lipids	D	ED-1	[207]	High individual variability of CD1a induction after G4. Lipoproteins (VLDL > LDL > HDL) and PPAR*γ* activation reduce the number of G4-induced CD1a**^+^** cells.
	Lysophosphatidic acid (LPA)	D	ED-1	[208]	*Mechanism: *LPA is a potent natural ligand for PPAR*γ.*
	Oxidized Phospholipids	D	ED-1	[209]	Oxidized phospholipids (generated during inflammation) down-regulate CD1a/b/c and block histone modifications required to activate mDCs.

Malignant cell products	Hepatoma cell supernatant	D	ED-1	[210]	CD1a down-regulation by hepatoma but not normal liver cell supernatants. Induction of Treg *Mechanism:* possibly IL-10-dependent.
	Human renal cell carcinoma lines	D	OED	[211]	From CD34**^+^** progenitor cells: severe inhibition of CD1a and APC function of induced CDs. *Mechanism*: possibly due, at least in part, to IL-6 and macrophage colony stimulating factor.
	Leukemia cell supernatant	D	*ED-1*	[212]	Supernatant of K562, HL-60 and DAUDI on CD1a expression. *Mechanism*: at least in part, due to IL-1*β* secreted by MOs in response to leukemic cell products.
	Melanoma cell supernatant	D	ED-1 IvDC	[213]	CD1a/b/c. *Mechanism:* IL-10 release induction *in vivo* reduced CD1-positive cells in metastatic melanoma.
		D	OED	[214]	LC generated *in vitro* from cord blood CD34**^+^** progenitors are CD1a-deficient when cultured with melanoma cells in a transwell design setting.
	Supernatant from primary or long-term cultured tumor cells	D	ED-1	[215]	Reduction of CD1a by supernatant of tumor cell lines was much less active respect to supernatants of primary tumors. Similar results obtained with CD34**^+^** progenitor-derived DCs. *Mechanism*: at least in part mediated by PGE2 released by primary tumor cells.

Nucleotides	cAMP, cGMP	D	ED-1	[216]	cAMP increase was mimicked by the adenylate cyclase activator forskolin or cAMP analog 8 bromo-cAMP; cGMP increase was mimicked by 8 bromo-cGMP; increase of both was induced by PDE inhibitor IBMX. Down regolation of CD1a is followed by impairment of LPS-induced mDC function.

Serum and serum components	Human serum	D	ED-1	[217]	Human serum lipids: impairment of CD1a/b/c transcription. Reduced induction of CD1c-restricted T-cell responses.* Mechanism: *PPAR*γ* activation.
				[218]	Human serum: *Mechanism: *PPAR*γ* activation and IL-10 induction.
				[219]	Autologous serum (iDCs from MOs or from CD34**^+^** precursors).
	IgG	D	ED-1	[220]	Down-regulation of CD1a/b/c and upregulation of CD-1d transcripts. *Mechanism*: IgG-mediated activation of Fc*γ* receptor Fc*γ*RIIa (CD32a).
		D	ED-1 ED-4	[221]	This study starts from the observation that intravenous immunoglobulin attenuates MS.
	*β*2-microblobulin	D	ED-1 ED-4	[222]	Down-regulation of CD1a and mDC function. *Mechanism*: inhibition of MAPK, ERK, MEK, and NF-*k*B, and activation of STAT3.

*Physical agents*					

Ultraviolet light	UVAI (340–400 nm)	D	IvDC	[223]	Decrease of CD1a**^+^** LC in a epidermis 3 days after ultraviolet exposure.
	UVB	D	IvDC	[224]	UV irradiation induces CD1a**^+^** LC down-regulation and IL-10 induction* in vivo* in skin. This is prevented* in vivo* by Zn-containing or octylmetoxy cinnamate sunscreen preparations.
		D	IvDC	[225]	CD1a**^+^** Langerhans cell loss after exposure of human epidermis and dermis to UVB, accompanied by infiltration with IL-10 producing macrophages.
		D	IvDC	[226]	Organ culture *in vitro* of human cornea (immunohistochemistry): low-dose UVB (100 mJ/cm^2^) decreases HLA-DR and CD1a expression of organ-cultured human corneas and induces moderate corneal injuries, and might be useful for preventing allograft rejection.

^a^Evaluation of CD1a expression if not otherwise specified: U: upregulation; D: down-regulation.

^b^Experimental design code (see Figure 2).

^c^Reference number.

**Table 5:** Influence exerted by infectious agents or microorganism products on group 1 CD1 antigen expression.

Infectious Agent	Agent	CD1^a^	ED^b^	Ref.^c^	Observations
Bacteria	*Helicobacter pilori E. coli*	D	ED-1	[227]	*In vitro* exposure to paraformaldehyde-fixed bacteria. IL-10 independent.
	*Helicobacter pilori* (*in vivo*)	U	IvDC	[228]	*In vivo* detected by antral biopsies (lamina propria): increased CD1a and CD1b in infected children with respect to normal subjects. Expression of local immune responses.
	*Propionibacterium acnes*	U	ED-2	[229]	Heat-killed bacteria added on day 6 to iDCs of pts with acne vulgaris.
	Various	U	OED	[230]	Increased CD1a expression in* in vitro* generation of mDCs from MS pts with bacterial infections versus MS pts without infections.

Chlamydia	*Chlamydia trachomatis*	U	IvDC	[231]	Myeloid DCs collected from cervical mucosa of chlamydia-infected woman show myeloid DCs with increased CD1a expression with respect to that of healthy women.

Mycoplasma	*Mycoplasma* present in cell culture supernatant	D	ED-1	[232]	If mycoplasma is removed, culture supernatants are no more able to down-regulate CD1a.

Protozoa	*Leishmania donovani *	D	ED-1	[233]	*L. donovani *infection *in vitro* impairs induction of CD1a/b/c expression in terms of gene transcript and protein.
	*Leishmania amazonensis*	D	ED-1 ED-4	[234]	Leishmania or soluble Leishmania antigen inhibited CD1a expression, but did not prevent further DC maturation toward CD83**^+^** mDCs.
	*Leishmania donovani and Leishmania major*	D	OED	[235]	*In vitro* G4-induced iDCs were infected with *L. donovani* or *L. major* on day 7 and tested for CD1a/b/c/d expression 8 h later. Down-regulation of mainly Group I CD1 molecules at the transcriptional (qRT-PCR) and surface expression levels was detected.
	*Toxoplasma gondii*	NC	OED	[236]	*In vitro* infection of untreated MOs with *T. gondii* does not induce CD1a.

Helminthes	*Necator Americanus*	D	OED	[237]	*In vitro* G4-induced CD1a in mDCs is lower when MOs were obtained from infected pts versus normal donors.
	*Echinococcus granulosus *	D	ED-1 ED-4	[238, 239]	Hydatid cyst components (AgB- and SHF) down-regulate CD1a and further prevent IL-12 production, increasing IL-10 release.

Viruses *(for HIV& HTLV-I see text) *	Human Herpes Virus-8 (alive or UV-inactivated)	D	ED-1	[240]	Reduced mDC activity and sixfold reduction in IL-12 (p70) production with consequent impairment of T-cell-mediated responses.
	Cytomegalovirus	D	ED-1	[241]	CD1a assay at day 7, before adding LPS (to evaluate iDC CD1a expression). Down-regulation of CD1a occurs also with UV-inactivated virus. Moreover, HCMV-infected mDCs were unable to induce a T-cell response, in line with the immunodepressive effects of HCMV infection.

*Micro-Organism products*					

*Bordetella pertussis *	Pertussis toxin	D	ED-1	[242]	Selective suppression of CD1a (mRNA and protein) but not of CD1b and CD1c. LPS-induced mDCs are functionally normal.

*Candida albicans*	Supernatant	D	ED-1	[243]	*C. albicans* supernatants contain a glycoprotein termed “Secretory IL-12 Inhibitory Factor”, able to down-regulate CD1a expression and IL-12 production by iDCs and DCs.

Dengue	Dengue-2 (live attenuated vaccines)	U	ED-2	[244]	DC maturation step 2 was attained with live attenuate vaccine LAV2 or DEN2 without adding LPS. Heat-inactivated virus was used as a negative control for virus infection. CD1a assay was performed after incubation at 32°C for 48 h.

Malaria	Hemozoin (malaria pigment)	D	ED-1 ED-4	[245]	*Mechanism*: increased PPAR*γ* expression (qRT-PCR) following hemozoin-induced activation in MOs.
	Atypical Membrane Antigen-1 (AMA-1)	U	ED-4	[246]	mDCs of *P. vivax* infected pts show lower CD1a expression than that of mDCs developed *in vitro* from MOs of noninfected controls. *In vitro* exposure to AMA-1 increases CD1a levels in mDCs developed *in vitro* from MOs of infected donors.

Mycotoxins	T-2 toxin	D	ED-1	[247]	Strong inhibition.

*Staphylococcus aureus *	Staphylococcus superantigen	U	IvDC	[248]	CD1a**^+^** cell number in the epidermis was significantly higher in the lesional skin with respect to that in non-lesional skin from atopic dermatitis pts or to that in the skin from normal donors.

Various bacteria	LPS	D	ED-1	[249]	LPS (from *Salmonella*) was added to MOs+G4 culture on day 0 instead of on day 5–9, as usually used for inducing iDC maturation to mDCs. *Mechanism:* in part by induction of IL-10, and mostly by MAPKp38 activation followed by ERK and NF-*k*B inactivation.

^a^Evaluation of CD1a expression if not otherwise specified: U: upregulation; D: down-regulation.

^b^Experimental design code (see Figure 2).

^c^Reference number.

## Abbreviations

Ac2SGL: Acylated sulfoglycolipid
APC: Antigen-presenting cells
ASA: Acetylsalicylic acid
ATF-2: Activating transcription factor
ATRA: All trans-retinoic acid
AZT: Zidovudine
BCG: Bacillus Calmette-Guerin
BDP: Beclomethasone dipropionate
CRE: cAMP response element
CREB-1: CRE-binding protein
DC: Dendritic cells
DEX: Dexamethasone
ERK: Extracellular signal-regulated kinases
G4: GM-CSF + IL-4
GA: Glatiramer acetate
GM-CSF: Granulocyte-macrophage colony stimulating factor
GST: Gold sodium thiomalate
HDAC: Histone deacetylases
iDC: Immature dendritic cells
IFN*:*: Interferon
IL: Interleukin
LC: Langerhans cells
LPS: Lipopolysaccharides
MAPK: Mitogen-activated protein kinase
mDC: Mature dendritic cells
MDR: Multidrug resistance
MIN: Minocycline
MM: Multiple myeloma
MMF: Monomethyl-fumarate
MOs: Monocytes
MRP1: Multidrug resistance protein 1
MS: Multiple sclerosis
MTP: Microsomal triglyceride transfer protein
NFA: Niflumic acid
NSAID: Nonsteroidal anti-inflammatory drugs
PBMN: Peripheral blood mononuclear cells
PPAR: Peroxisome proliferator-activated receptor
pts: Patients
RA: Rheumatoid arthritis
TCR: T-cell receptor
TLR: Toll-like receptors
TNF: Tumor necrosis factor.
